# Development and validation of a CT radiomics and clinical feature model to predict omental metastases for locally advanced gastric cancer

**DOI:** 10.1038/s41598-023-35155-y

**Published:** 2023-05-25

**Authors:** Ahao Wu, Changlei Wu, Qingwen Zeng, Yi Cao, Xufeng Shu, Lianghua Luo, Zongfeng Feng, Yi Tu, Zhigang Jie, Yanyan Zhu, Fuqing Zhou, Ya Huang, Zhengrong Li

**Affiliations:** 1 Department of Digestive Surgery, Digestive Disease Hospital, The First Affiliated Hospital of Nanchang University, Nanchang, 330006 Jiangxi Province China; 2 Department of Gastrointestinal Surgery, The Second Affiliated Hospital, Nanchang University, Nanchang, 330006 Jiangxi Province China; 3 Medical Innovation Center, The First Affiliated Hospital of Nanchang University, Nanchang, China; 4 Department of Pathology, The First Affiliated Hospital of Nanchang University, Nanchang, 330006 Jiangxi Province China; 5 Department of Radiology, The First Affiliated Hospital, Nanchang University, Nanchang, 330006 Jiangxi Province China; 6 Department of Radiology, The Second Affiliated Hospital, Nanchang University, Nanchang, China

**Keywords:** Cancer, Oncology, Risk factors

## Abstract

“”We employed radiomics and clinical features to develop and validate a preoperative prediction model to estimate the omental metastases status of locally advanced gastric cancer (LAGC). A total of 460 patients (training cohort, n = 250; test cohort, n = 106; validation cohort, n = 104) with LAGC who were confirmed T3/T4 stage by postoperative pathology were continuously collected retrospectively, including clinical data and preoperative arterial phase computed tomography images (APCT). Dedicated radiomics prototype software was used to segment the lesions and extract features from the preoperative APCT images. The least absolute shrinkage and selection operator (LASSO) regression was used to select the extracted radiomics features, and a radiomics score model was constructed. Finally, a prediction model of omental metastases status and a nomogram were constructed combining the radiomics scores and selected clinical features. An area under the curve (AUC) of the receiver operating characteristic curve (ROC) was used to validate the capability of the prediction model and nomogram in the training cohort. Calibration curves and decision curve analysis (DCA) were used to evaluate the prediction model and nomogram. The prediction model was internally validated by the test cohort. In addition, 104 patients from another hospital's clinical and imaging data were gathered for external validation. In the training cohort, the combined prediction (CP) model (AUC 0.871, 95% CI 0.798–0.945) of the radiomics scores combined with the clinical features, compared with clinical features prediction (CFP) model (AUC 0.795, 95% CI 0.710–0.879) and radiomics scores prediction (RSP) model (AUC 0.805, 95% CI 0.730–0.879), had the better predictive ability. The Hosmer–Lemeshow test of the CP model showed that the prediction model did not deviate from the perfect fitting (*p* = 0.893). In the DCA, the clinical net benefit of the CP model was higher than that of the CFP model and RSP model. In the test and validation cohorts, the AUC values of the CP model were 0.836 (95% CI 0.726–0.945) and 0.779 (95% CI 0.634–0.923), respectively. The preoperative APCT-based clinical-radiomics nomogram showed good performance in predicting omental metastases status in LAGC, which may contribute to clinical decision-making.

## Introduction

Gastric cancer (GC) was one of the most common malignant tumors worldwide. In 2020, There were approximately 1.09 million new cases and 770,000 deaths worldwide, with GC morbidity and mortality ranking fifth and fourth, respectively^1^. Most GC patients were in the advanced stage once diagnosed because there were no obvious clinical symptoms in early GC patients^2^. The primary treatment for locally advanced gastric cancer (LAGC) patients was radical gastrectomy. However, the benefits of resecting the omentum during surgery were still inconclusive. The Japanese GC treatment guidelines (5th edition) note that for cT1–T2 tumors, the omentum of more than 3 cm away from the gastroepiploic arcade could be preserved^3^. However, there was no clinical benefit from omentectomy with advanced GC. There was no significant difference in the recurrence rate or long-term survival between the omentum preservation group and the omentectomy group^4,5^. In contrast, radical gastrectomy with omentum preservation had the advantages of a short operation time, less intraoperative bleeding, and fewer postoperative complications^6,7^. Especially in obese patients, the omentum was large and difficult to operate on during laparoscopic gastrectomy surgery, while omentum preservation can overcome these technical difficulties^4^. In conclusion, resection of the omentum in patients with LAGC may prolong operative time and increase bleeding, whereas patients with preserved omentum had the same survival benefits as those with resected omentum. It is now generally accepted that patients with LAGC without omental metastases can preserve the omentum during radical surgery^4,8,9^. As a result, knowing how to accurately identify omental metastases status before surgery is critical for performing radical surgery with preserved omentum.

The gold standard for the diagnosis of omental metastasis was the pathological results after surgery, but it was difficult to implement for patients with omentum preservation. Micro omental metastases that were too small to be seen with the naked eye were difficult to detect with a routine preoperative examination. Computed Tomography (CT) was a commonly used imaging method for the diagnosis of GC. It had the advantages of high spatial resolution, non-invasiveness, and strong image-processing technology support. The differentiation degree, pathological type, TNM staging, and evaluation of chemotherapy efficacy of GC were analyzed^10–12^. However, CT showed poor sensitivity and specificity for detecting micro omental metastases^13^. The detection of potential omental metastases remains a challenge for surgeons^14^. Therefore, there is an urgent to develop a novel, non-invasive and accurate preoperative detection technique, as an auxiliary diagnostic tool for omental metastases status in advanced GC.

Radiomics, which has attracted increasing attention in recent years, was the process of converting medical images into high-dimensional, mineable data through high-throughput extraction of quantitative features, followed by data analysis to obtain decision support^15^. Through quantitative features of regions of interest that were extensively extracted^16^, radiomics can noninvasively detect tumor biology, distinguish subtle differences that cannot be identified by human eyes, and quantify tumor heterogeneity^17^. The quantitative features extracted from radiological images also can reflect biological information such as cell morphology, gene, and molecular expression, and tumor heterogeneity to a certain extent^18^. The application of preoperative CT radiomics in advanced GC to predict peritoneal metastasis had preliminarily demonstrated the predictive value of CT radiomics in peritoneal metastasis^19,20^. In addition, radiomics features were completely different and complementary to clinical data^21^, so the combination of radiomics features and clinically relevant data can produce an accurate and reliable evidence-based clinical decision support system^22,23^. This provided us with the possibility to use radiomic features and clinical data to predict omental metastases status in LAGC patients.

Therefore, we hypothesized that radiomics might be beneficial in predicting omental metastases status. the main purpose of our study was to use preoperative CT radiomics features and clinical data to establish a predictive model for omental metastasis and to draw an individualized nomogram. Validation of the predictive model and analysis of the clinical benefit was performed.

## Materials and methods

### Patients and study design

**Figure 1:**
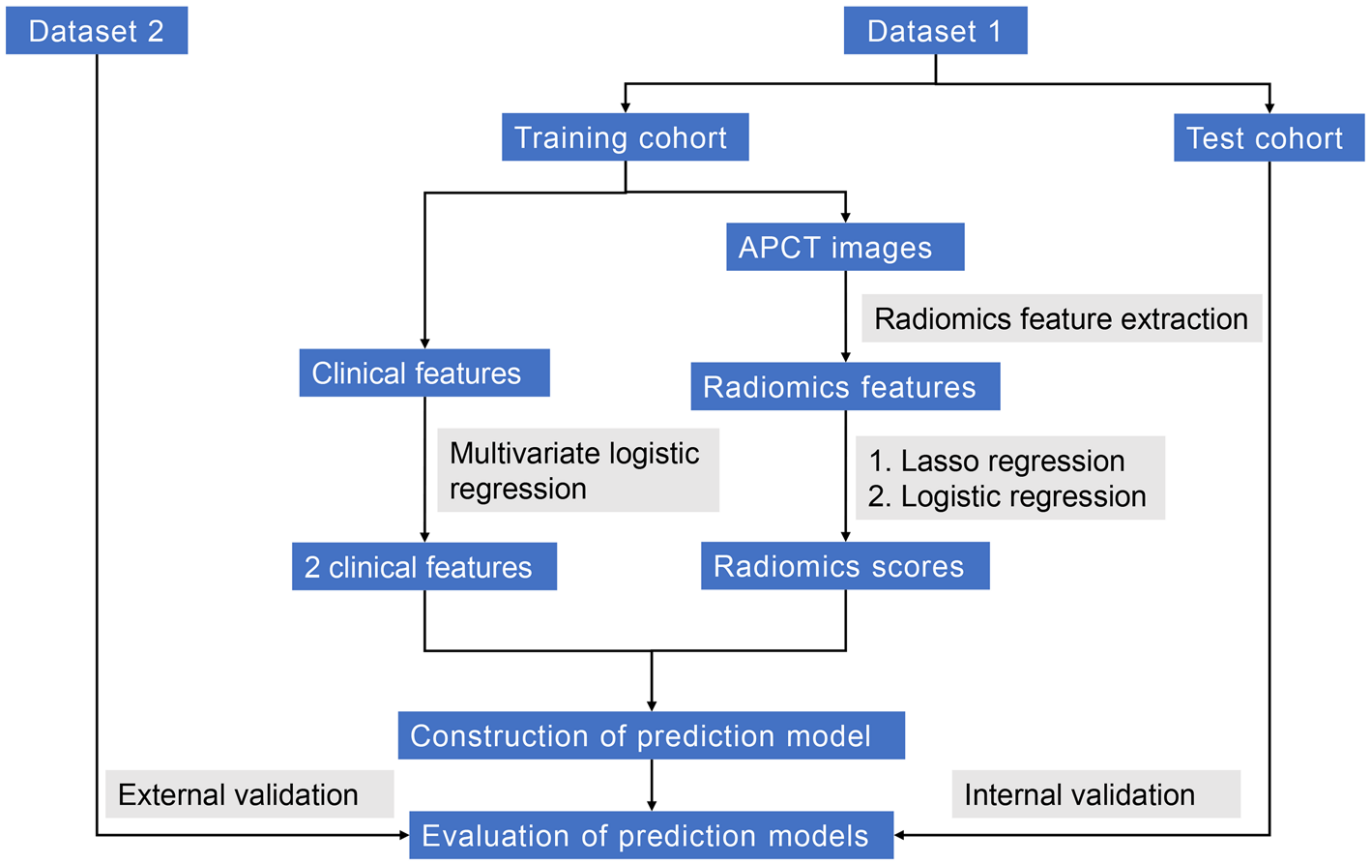
Flow of the study design.

A total of 460 patients with pathologically proven LAGC were continuously enrolled from April 28, 2020, to June 30, 2021, and the collected data included clinical features and arterial phase computed tomography (APCT) images. Of which 356 patients from the First Affiliated Hospital of Nanchang University were randomly divided into a training cohort and a test cohort in a ratio of 7:3. In addition, 104 patients from the Second Affiliated Hospital of Nanchang University served as the validation cohort. The clinical features included age, sex, body mass index (BMI), carcinoembryonic antigen (CEA), carbohydrate antigen 19-9 (CA19-9), cancer antigen 125 (CA125), neutrophil-to-lymphocyte ratio (NLR), platelet-to-lymphocyte ratio (PLR), albumin, CT-reported LN status, tumor location, tumor size, clinical T stage, clinical N stage, and Borrmann classification. The inclusion criteria were as follows: (1) a preoperative enhanced abdominal CT examination within 2 weeks; (2) omentectomy during radical gastrectomy in patients with LAGC; (3) histologically confirmed primary gastric adenocarcinoma; and (4) postoperative pathology confirmed to be T3/T4 stage. The exclusion criteria were as follows: (1) patients with incomplete clinical data; (2) postoperative pathology confirmed to be T1/T2 stage; (3) the image quality was insufficient for diagnosis due to artifacts or poor distention on CT images; and (4) neoadjuvant radiotherapy was performed before surgery. The flow of the study design was shown in Fig. 1. The Medical Research Ethics Board of the First Affiliated Hospital of Nanchang University, China, waived the informed consent in the main manuscript [The Ethics Board approval number: (2022)CDYFYYLK(08-007)]. All methods were performed in accordance with the relevant guidelines and regulations.

### CT examination and acquisition parameters

All patients were required to fast for at least 4 h. Racanisodamine hydrochloride (654-II) 20 mg was administered intramuscularly to reduce their gastrointestinal peristalsis 10–15 min before the CT examination, then patients drank approximately 1000 mL of water to distend their stomach. Patients were asked to hold their breath while the CT examination was performed using a 64-slice spiral CT (SOMATON sensation64, Siemens Healthineers, Germany). Patients were infused with 1.5 mL/kg of nonionic contrast material (iohexol, Yangzi River Pharmaceutical Group, Jiangsu, China; iodine concentration: 300 mg/mL) into the antecubital vein using a pump injector at a speed of 3.0 mL/s. Following a routine unenhanced scan, a contrast-enhanced CT was performed 25 to 30 s (arterial phase) and 60 s (venous phase). The acquisition parameters were as follows: 120 kVp; tube current, auto; rotation time, 0.5 s; detector collimation, 64 × 0.625 mm or 192 × 0.625 mm; field of view, 350 × 350 mm; pitch, 0.656 and 0.7, respectively; matrix, 512 × 512. The raw data were reconstructed with a 3-mm section thickness for the routine axial CT images.

### Radiomics features acquisition

#### Region of interest (ROI) segmentation

APCT images were downloaded and saved in Digital Imaging and Communications in Medicine (DICOM) format from the radiology center. ROI in the CT images can be determined according to the electronic gastroscope and the CT report. ROI segmentation was performed by using *3D Slicer* software (version 4.11.20210226). The ROIs of all subjects were manually segmented by one radiologist with more than 5 years of experience in abdominal radiology. The contours of the tumor were drawn carefully along the tumor boundary of each CT slice. Another senior radiologist with 10 years of experience in abdominal radiology randomly selected 50 patients (25 cases of omentum metastasis and 25 cases of omentum nonmetastatic) for ROI segmentation to evaluate the interoperator variability. The segmented CT image files were saved in Nearly Raw Raster Data (NRRD) format.

#### Radiomics features extraction

Using plug-in *pyradiomics* in *3D Slicer*, radiomics features were extracted from the CT images of all subjects. Four types of radiomics features can be extracted from ROIs: (1) shape and size features, reflecting the three-dimensional size and shape of the tumor; (2) first-order statistical features, reflecting the distribution characteristics of the voxel intensity in the selected region; (3) texture features, describing the spatial distribution of the pattern or voxel intensity, calculated by gray-level co-occurrence matrix (GLCM) and gray-level run-length matrix (GLRLM); and (4) wavelet features, wavelet decompositions of first-order statistical features and texture features.

#### Feature selection and radiomics score construction

**Figure 2:**
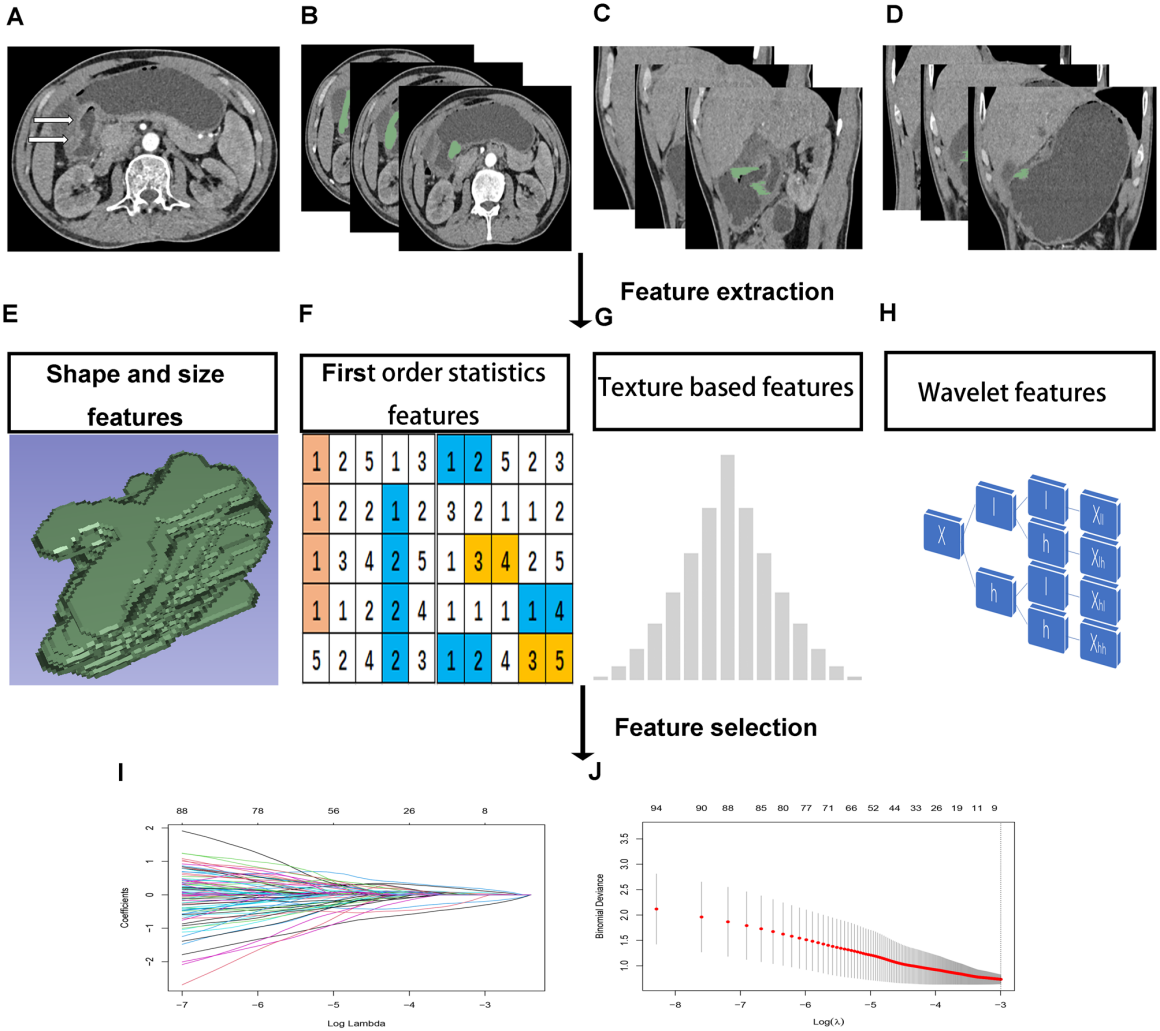
Flow of radiomics features acquisition. (**A**) Detecting the location of the tumor; (**B–D**) Segmentation of ROI, in the transverse, coronal and sagittal planes, respectively; (**E–H**) Feature extraction; (**I**, **J**) Features selected by LASSO regression.

The intraclass correlation coefficient (ICC) value of each radiomics feature was calculated, and features with ICC values higher than 0.75 were considered reliable and stable and were retained. The least absolute shrinkage and selection operator (LASSO) regression was used to select the radiomics features. Each feature has a related regression coefficient, and with a continuous increase in the λ value, some regression coefficients of the features continually decrease and trend toward 0. Features with nonzero coefficients were selected as valuable predictors to construct a radiomics score model. The selected radiomics features were analyzed by multivariate logistic regression to construct the most appropriate radiomics score model. The correlation coefficients and constant of the model were calculated, and the radiomics score formula was inferred. It was worth noting that the feature selection by LASSO regression and the construction of the radiomics score model were all from the date of the training cohort. Then, the feature scores of all patients were calculated according to the radiomics score formula. The radiomics feature acquisition process was shown in Fig. 2.

### Construction and evaluation of the nomogram

Multivariate regression was performed on radiomics scores and clinical features to screen for independent risk factors for omental metastases. The radiomics scores and selected clinical features of the training cohort were used to construct the radiomics score prediction (RSP) model, the clinical feature prediction (CFP) model, and the combined prediction (CP) model of the radiomics features combined with the clinical features and to draw an individualized nomogram to predict omental metastases. The predictive ability of the three prediction models was compared, and the area under the curve (AUC) value of the receiver operating characteristic (ROC) curve, net reclassification improvement (NRI), calibration curve, concordance index (C-index) and decision curve analysis (DCA) were used to evaluate the prediction models. Then, the prediction model was verified with test and validation cohort data.

### Statistical analysis

Statistical analysis was performed with SPSS software (version 21.0), R software (version 4.13), and Python software (version 3.7). Qualitative variables were presented as frequencies, and the differences between qualitative variables were compared by the chi-square test. Normally distributed variables were expressed as the mean ± SD. A t-test was used to compare the differences among the normally distributed variables. A two-tailed p-value less than 0.05 was considered statistically significant. ICC was used to evaluate the interoperator variability of the radiomics features. A scatter plot was used to illustrate the difference in the radiomics scores in both cohorts. The predictive ability of the omental metastasis prediction model in both cohorts was evaluated with ROC curves. The difference in the AUC between different prediction models was assessed with the Delong test, and the NRI was used to compare the CP model with the CFP model to evaluate the improvement degree of the CP model. The calibration curve and C-index were used to evaluate the fitting of the prediction model. Finally, the DCA of the prediction models was performed to evaluate the clinical usefulness of the prediction models by calculating the net benefits in the training and test cohorts.

### Ethics statement

The studies involving human participants were reviewed and approved by the Ethics Committee of the First Affiliated Hospital of Nanchang University. The Medical Research Ethics Board of the First Affiliated Hospital of Nanchang University, China, waived the informed consent in the main manuscript [The Ethics Board approval number: (2022)CDYFYYLK(08–007)].

## Results

### Clinical characteristics

Among the 460 eligible patients, 406 had no omental metastases, and 54 had omental metastasis. The incidence of omental metastasis was 11.7%. According to a proportion of 7:3, 356 patients from the First Affiliated Hospital of Nanchang University were randomly divided into a training cohort (n = 250) and a test cohort (n = 106). The average age of patients was 62.01 ± 10.69 years old in the training cohort, 220 patients had no omental metastasis, and 30 patients had omental metastasis. The average age of patients was 61.75 ± 10.75 years old in the test cohort, 95 patients had no omentum metastasis, and 11 patients had omentum metastasis. The features of both cohorts were compared, and the results showed that there was no significant difference (Table 1), ensuring the reliability of the test cohort as the internal validation data. The average age of patients was 65.09 ± 10.72 years old in the validation cohort, 91 patients had no omentum metastasis, and 13 patients had omentum metastasis. Detailed clinical information of the validation cohort was shown in Additional Table 1.

**Table 1:** Characteristics of LAGC patients in the training cohort and test cohort.

Characteristics	Training cohort (n = 250)	Test cohort (n = 106)	*P* value
Age (years)	62.01 ± 10.69	61.75 ± 10.75	0.83
BMI (Kg/m^2^)	22.21 ± 3.01	22.00 ± 3.02	0.54
NLR	3.25 ± 3.76	2.71 ± 1.76	0.15
PLR	182.93 ± 93.98	172.51 ± 101.73	0.34
Albumin (g/L)	39.05 ± 4.82	39.24 ± 4.64	0.72
Tumor size (cm^2^)	22.93 ± 22.68	21.59 ± 28.83	0.63
Omental metastases			0.97
No	220	95	
Yes	30	11	
Gender			0.18
Male	183	71	
Female	67	35	
CT-reported LN status			0.10
LN (−)	124	44	
LN (+)	126	62	
CEA			0.67
Normal	207	85	
Abnormal	43	21	
CA125			0.43
Normal	233	101	
Abnormal	17	5	
CA19-9			0.98
Normal	187	81	
Abnormal	63	25	
Borrmann classification			1.00
I	8	3	
II	80	35	
III	154	64	
IV	8	4	
Tumor location			0.38
Proximal third	33	16	
Middle third	72	19	
Distal third	141	69	
Complete stomach	4	2	
Clinical T stage			0.99
cT3	59	25	
cT4a	70	32	
cT4b	121	49	
Clinical N stage			0.44
N0	49	30	
N1	47	13	
N2	49	20	
N3a	57	26	
N3b	48	17	
Radiomics scores	− 2.41 ± 1.10	− 2.32 ± 1.10	0.49

*NLR* meutrophil-to-lymphocyte ratio, *PLR* platelet-to-lymphocyte ratio, *BMI* body mass index, *CT* computed tomography, *LN (−)* lymph note metastasis negative, *LN (*+*)* lymphnode metastasis positive, *CEA* carcinoembryonic antigen, *CA19-9* carbohydrate antigen 19-9, *CA125* cancer antigen 125.

### Radiomics features

**Figure 3:**
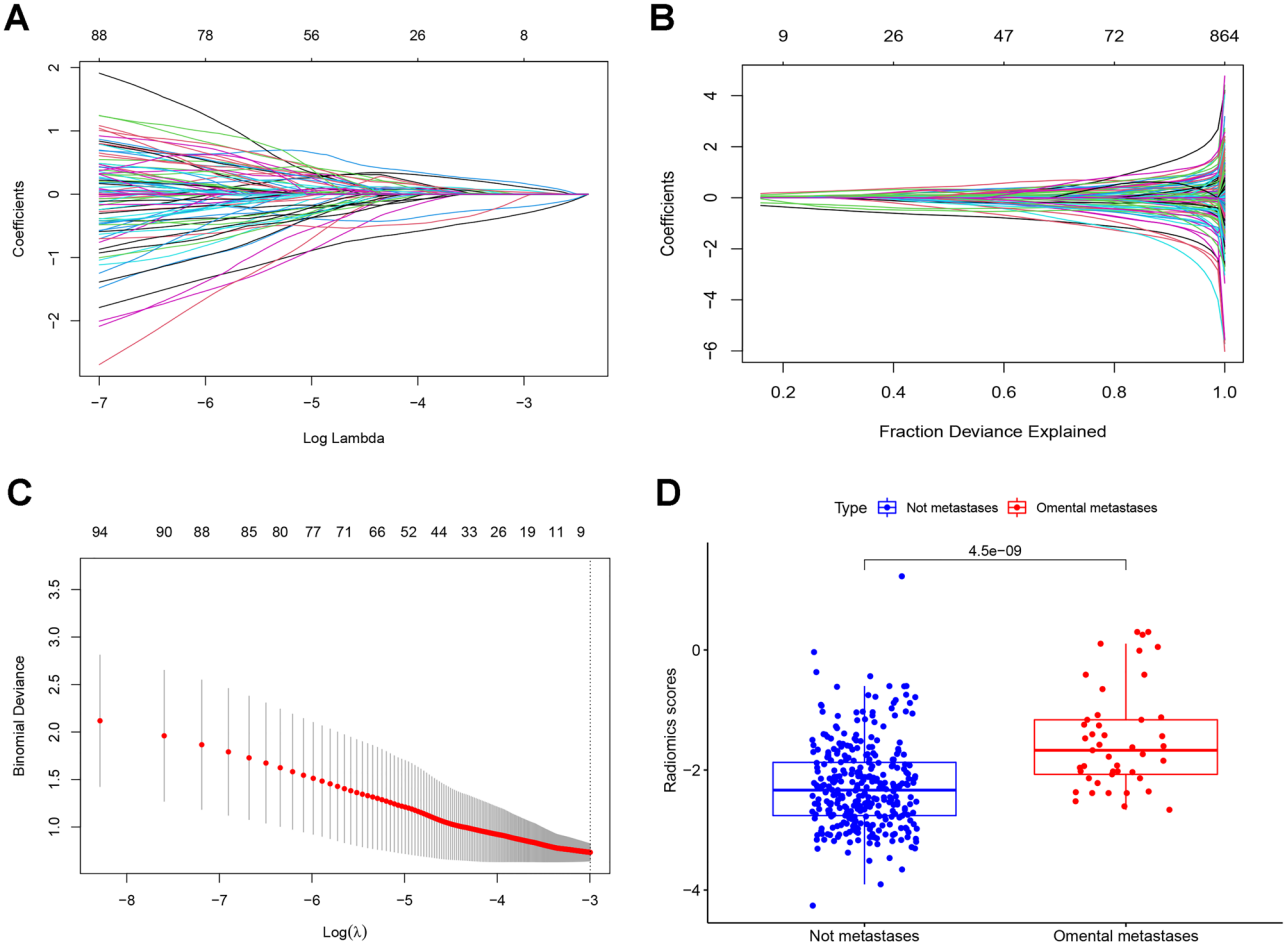
(**A**) The abscissa represents the λ value, and the ordinate represents the regression coefficient of the features. With the increase in λ value, the regression coefficient tends to be 0. (**B**) Fraction Deviance Explained; (**C**) The abscissa represents the λ value, the ordinate represents the mean square error, and the dotted line represents the number of features remaining while the mean square error was minimal. Finally, 7 radiomics features remained. (**D**) The difference in radiomics scores between the negative and positive groups of greater omentum metastasis (p < 0.001).

Altogether, 864 radiomics features were extracted from APCT images. The features with ICC values higher than 0.75 were considered to be stable, and finally, a total of 548 radiomics features were retained (Additional file 1), including 26 shape and size features, 17 first-order statistical features, 26 texture features, and 479 wavelet features. Radiomics features in the training cohort were selected by LASSO regression, and 9 features were significantly related to omental metastasis (Fig. 3A–C). The selected 9 features were analyzed by multivariate logistic regression, and only four features were chosen to construct the radiomics scores model, such as diagnostics Image original Mean (DIOM), original shape Maximum 2D Diameter Slice (OSMDS), original first order Kurtosis (OFOK) and wavelet LLH glcm Cluster Shade (WLGCS), respectively. The formula for the radiomics score calculation was as follows: radiomics score = 0.48 × DIOM + 0.5 × OSMDS + 0.047 × OFOK − 0.15 × WLGCS − 2.17. There was a significant difference in the radiomics scores between the omental metastasis-negative group and the omental metastasis-positive group (*p* value < 0.001) (Fig. 3D). The radiomics score of the positive group was significantly higher than that of the negative group.

### Construction of the nomogram

**Figure 4:**
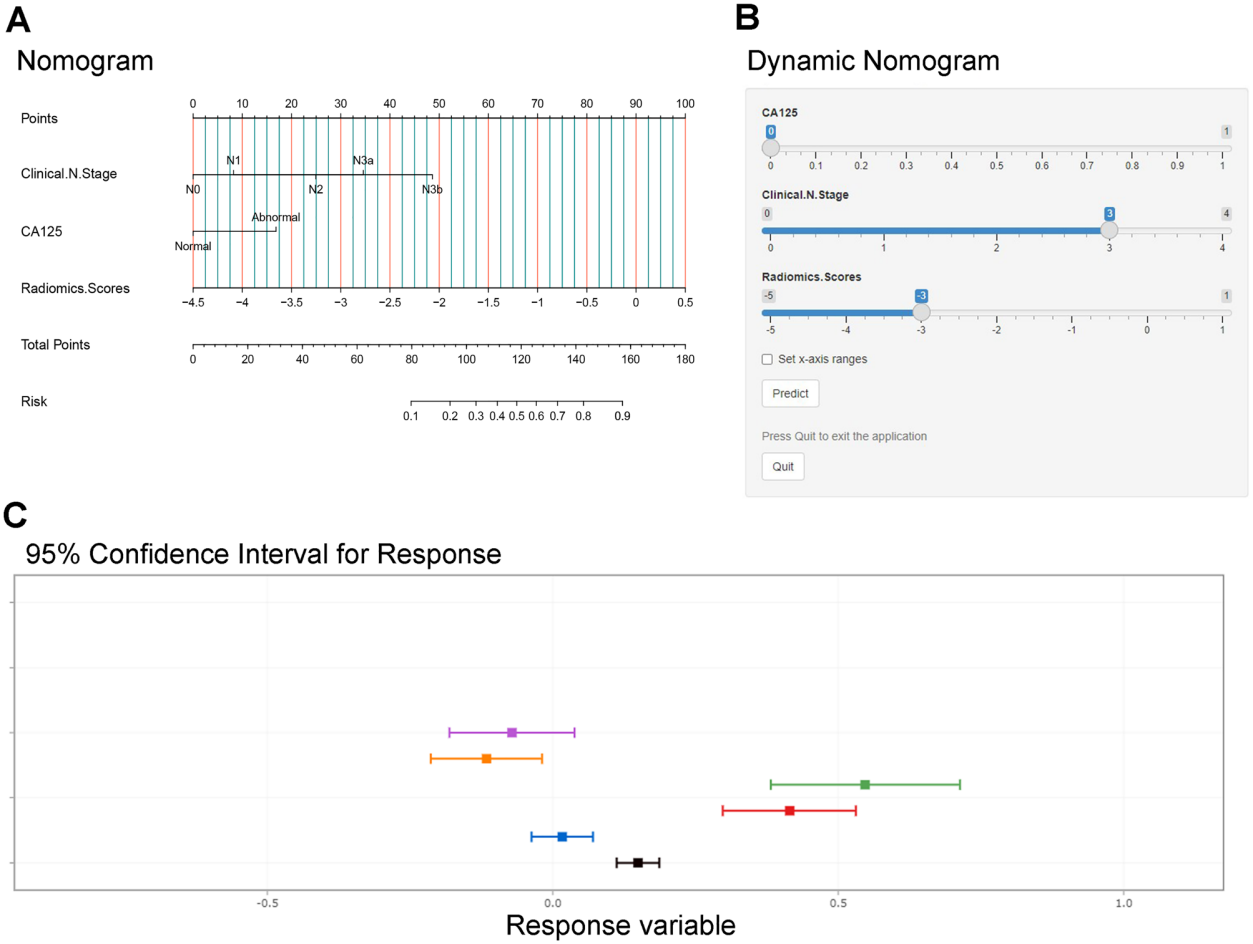
(**A**) Individualized nomogram for predicting greater omental metastasis of advanced GC. The score of each feature ranges from 0 to 100. The scale value of the risk of omental metastasis corresponding to the total scores is the predictive probability of omental metastasis (individual cases were shown in the Additional file 3). (**B**) Dynamic nomogram; (**C**) Dynamic nomogram predictions and 95% confidence interval.

Multivariable analysis revealed that the radiomics scores, CA125, and clinical N stage were significant independent factors for omental metastasis of advanced GC (Table 2). The RSP model, the CFP model (CA125, clinical N stage), and the CP model of the radiomics features combined with the clinical features were constructed. The nomogram of the CP model was built to predict the individual omental metastases status (Fig. 4A). Predictive nomograms of clinical features and radiomics scores were shown in Additional file 2. Considering the error caused by manual measurements, a dynamic nomogram was constructed (Fig. 4B,C), which did not need manual measurements, and after inputting the features of the patient, the probability of omental metastasis could be calculated directly at this website (https://wuahao123.shinyapps.io/DynNomapp/).

**Table 2:** Multivariate logistic regression analysis of risk factors of omental metastases.

Characteristics	OR	OR 95% L	OR 95% H	*P* value
NLR	0.95	0.80	1.12	0.53
PLR	1.00	0.99	1.00	0.29
Albumin	0.92	0.83	1.01	0.08
Age	0.98	0.94	1.02	0.23
BMI	0.97	0.84	1.12	0.66
Tumor size	1.00	0.99	1.01	0.83
Gender	2.49	0.749	8.34	0.14
CT-reported LN status	1.66	0.64	4.32	0.30
CEA	1.87	0.67	5.17	0.23
CA125	4.35	1.34	14.1	**0.014**
CA19-9	1.06	0.44	2.54	0.89
Borrmann classification (I)				0.19
II	3.07E + 08	0.00	–	1.00
III	4.12E + 08	0.00	–	1.00
IV	2.90E + 09	0.00	–	1.00
Tumor location (proximal third)				0.51
Middle third	3.28	0.64	16.90	0.16
Distal third	2.00	0.42	9.40	0.40
Complete stomach	0.00	0.00	–	1.00
Clinical T stage (T3)				0.27
cT4a	2.44	0.54	11.06	0.25
cT4b	3.02	0.79	11.62	0.11
Clinical N stage (N0)				**0.025**
N1	3.29	0.27	39.49	0.35
N2	5.88	0.63	54.64	0.12
N3a	5.44	0.56	52.65	0.14
N3b	17.13	2.007	141.46	0.008
Radiomics scores	4.93	2.81	8.66	** < 0.001**

“–” represent lack, *P* value bolded means statistically significant.

### Evaluation of prediction models

**Figure 5:**
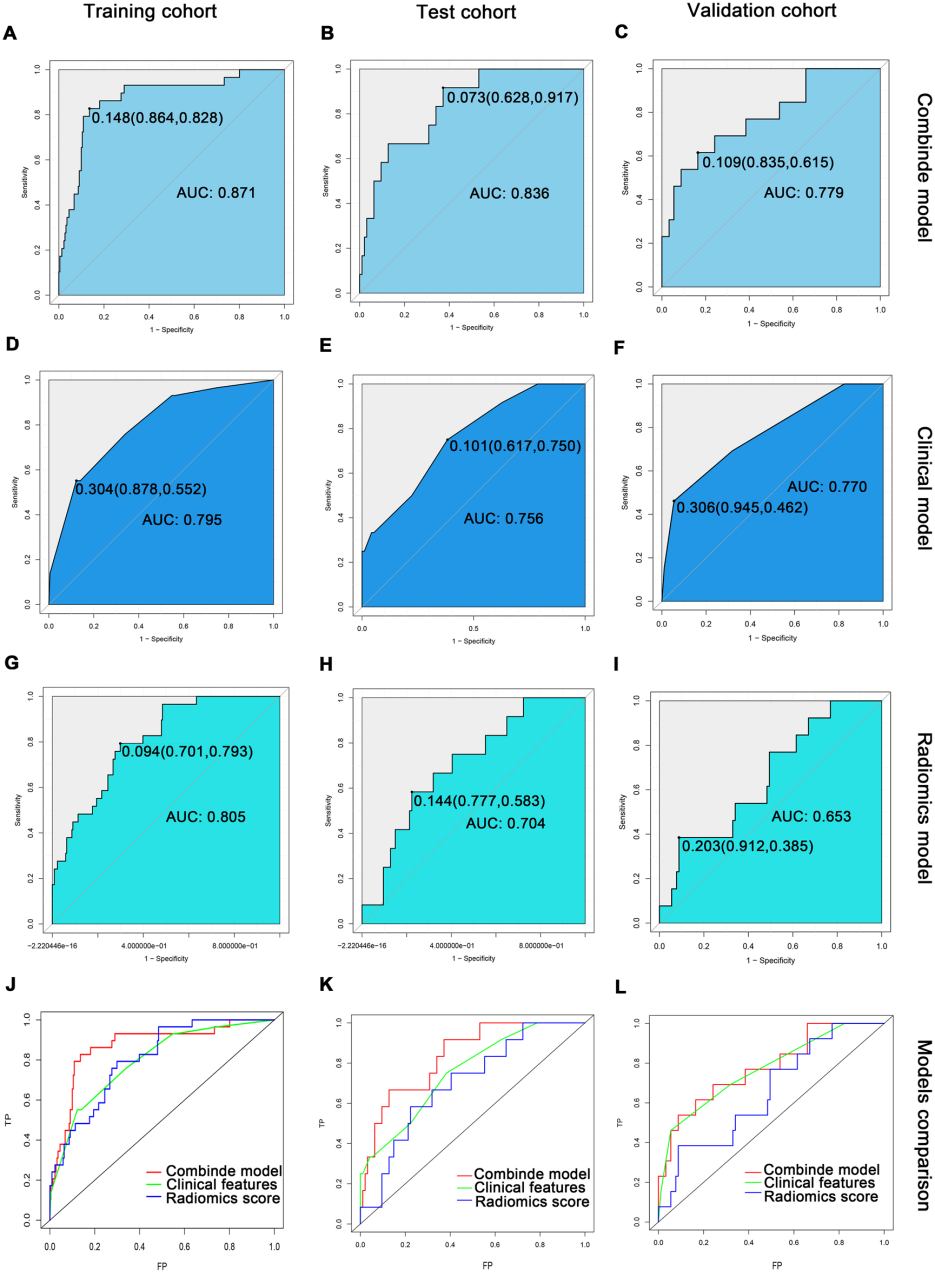
Training cohort (**A**) The ROC curve of the CP model (AUC 0.871, 95% CI 0.798–0.945); (**D**) The ROC curve of the CFP model (AUC 0.795, 95% CI 0.710–0.879); (**G**) The ROC curve of the RSP model (AUC 0.805, 95% CI 0.730–0.879); (**J**) The prediction ability of the three prediction models. Test cohort (**B**) The ROC curve of the CP model (AUC 0.836, 95% CI 0.726–0.945); (**E**) The ROC curve of the CFP model (AUC 0.756, 95% CI 0.614–0.897); (**H**) The ROC curve of the RSP model (AUC 0.704, 95% CI 0.558–0.850); (**K**) The prediction ability of the three prediction models. Validation cohort (**C**) The ROC curve of the CP model (AUC 0.779, 95% CI 0.634–0.923); (**F**) The ROC curve of the CFP model (AUC 0.770, 95% CI 0.624–0.916); (**I**) The ROC curve of the RSP model (AUC 0.653, 95% CI 0.498–0.808); (**L**) The prediction ability of the three prediction models.

In the training cohort, the AUC value of the CP model was 0.871 (95% CI 0.798–0.945) (Fig. 5A); the AUC value of the CFP model was 0.795 (95% CI 0.710–0.879) (Fig. 5D); the AUC value of the RSP model was 0.805 (95% CI 0.730–0.879) (Fig. 5G). The CP model had better predictive capability than the single CFP model (0.871 vs. 0.795; *p* value = 0.12) (Fig. 5J). The NRI value of the CP model was 0.262 (p value = 0.007) compared with the CFP model. The NRI value of the CP model was 0.197 (*p* value < 0.001) compared with the RSP model, indicating that the CP model improved the predictive capability of omental metastasis. In the test cohort, the AUC value of the CP model was 0.836 (95% CI 0.726–0.945) (Fig. 5B); the AUC value of the CFP model was 0.756 (95% CI 0.614–0.897) (Fig. 5E); the AUC value of the RSP model was 0.704 (95% CI 0.558–0.850) (Fig. 5H). In the test cohort, the CP model similarly exhibited satisfactory accuracy (the training cohort AUC = 0.871 vs. the test cohort AUC = 0.836; *p* value = 0.92). Although the CP model of the test cohort had a slightly lower AUC value than the training cohort, the difference was not statistically significant. The predictive capacity of the CP model was also better than that of the single CFP model. (NRI value = 0.177, *P* value = 0.03) (Fig. 5K). In the validation cohort, the omental metastatic prediction model retains had relatively good predictive power. The AUC values of the CP model were 0.779 (95% CI 0.634–0.923) (Fig. 5C); the CFP model was 0.770 (95% CI 0.624–0.916) (Fig. 5F); and the RSP model was 0.653 (95% CI 0.498–0.808) (Fig. 5I). A comparison of the AUC values for each prediction model was shown in Fig. 5L.

**Figure 6:**
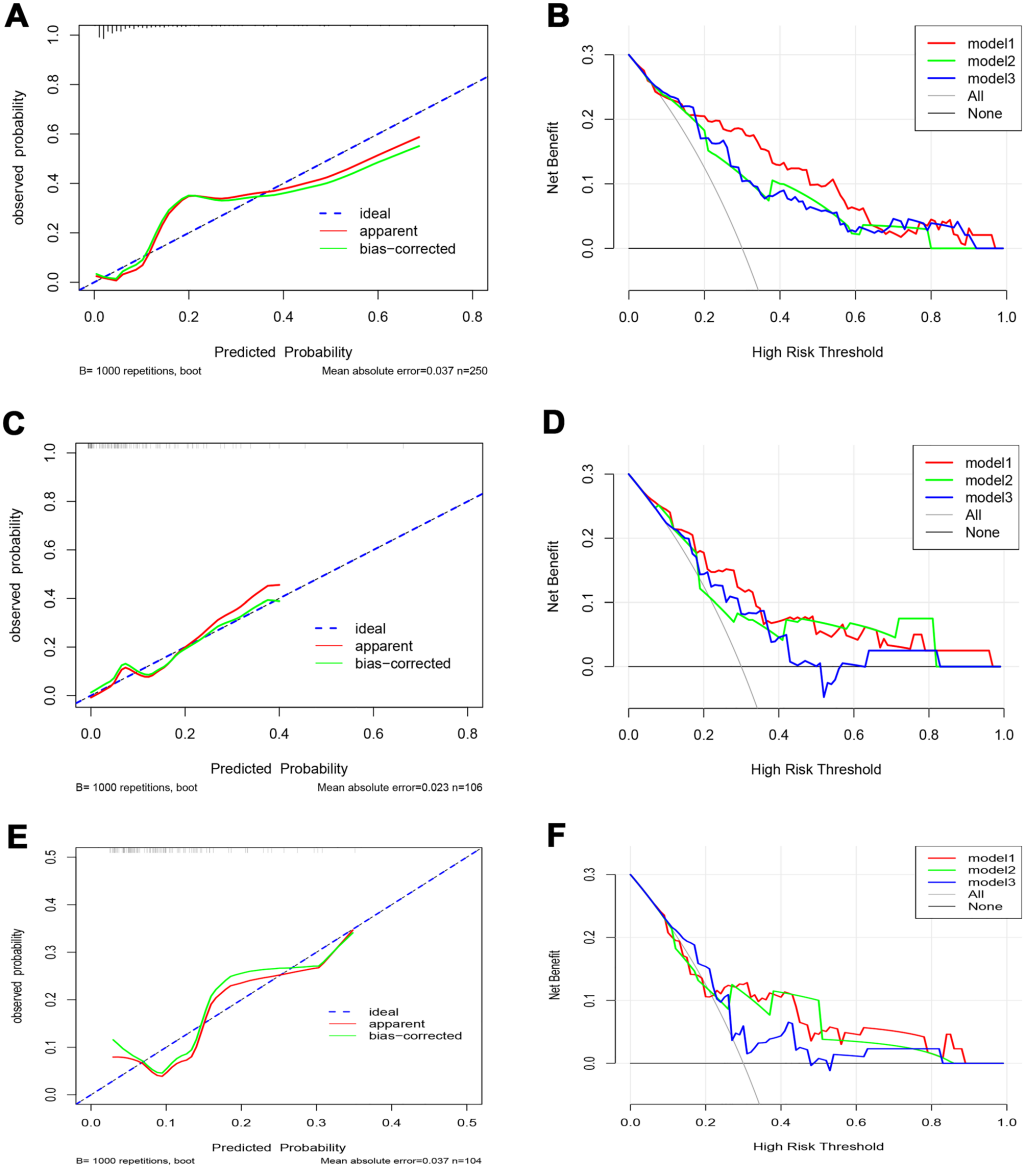
Training cohort (**A**) The calibration curve of the CP model in the training cohort. The abscissa represents the predictive probability of omental metastasis, and the ordinate represents the actual probability of omental metastasis. Ideally, the predictive probability is equal to the actual probability. The prediction probability of the CP model does not deviate much from the ideal curve. (**B**) DCA of the training cohort. Test cohort (**C**) Calibration curve of the CP model in the test cohort; similarly, the prediction probability of the CP model does not deviate markedly from the ideal curve. (**D**) DCA of the test cohort. Validation cohort (**E**) Calibration curve of the CP model in the validation cohort. (**F**) DCA of the validation cohort. The clinical net benefit of Model 1 was better than that of Model 2 and Model 3 in the training cohort, test cohort, and validation cohort. Model 1 = CP model; Model 2 = CFP model; Model 3 = RSP model.

In the training cohort, the calibration curve of the CP model was shown in Fig. 6A, and the Hosmer–Lemeshow test of the CP model showed that the model did not deviate from the perfect fit (p value = 0.893). The C-index of the CP model was 0.871, and the corrected C-index was 0.846, which showed that the CP model had high predictive accuracy. In the test cohort, the calibration curve of the CP model was shown in Fig. 6C, and the *p* value of the Hosmer–Lemeshow test was 0.448. This showed that the prediction model of omental metastasis constructed by the training cohort database was still applicable in the test cohort, and there was no overfitting or underfitting. The C-index of the CP model was 0.836, and the corrected C-index was 0.760 in the test cohort, indicating that the CP model still had good predictive accuracy in the test cohort. The omental metastasis prediction model constructed from the training cohort also performed relatively better results in the validation cohort (Fig. 6E). The C-index of the CP model was 0.720, and the corrected C-index was 0.665 in the validation cohort. The predictive capacity of each prediction model was shown in Table 3.

**Table 3:** Evaluation and comparison of model performance in the training cohort and test cohort.

Characteristics	AUC	Sensitivity	Specificity	C-index	Corrected C-index
Training cohort					
Model 1	0.871	0.828	0.864	0.871	0.846
Model 2	0.795	0.552	0.878	0.794	0.773
Model 3	0.805	0.793	0.701	0.805	0.805
Test cohort					
Model 1	0.836	0.917	0.628	0.836	0.76
Model 2	0.756	0.75	0.617	0.756	0.685
Model 3	0.704	0.583	0.777	0.704	0.699
Validation cohort					
Model 1	0.779	0.615	0.835	0.72	0.665
Model 2	0.77	0.462	0.975	0.716	0.682
Model 3	0.652	0.384	0.912	0.653	0.647

*Model 1* CP model, *Model 2* CFP model, *Model 3* RSP model.

In the training cohort, the decision curve analysis of the prediction models was shown in Fig. 6B. the abscissa represent the threshold probability and the ordinate represents the clinical net benefit; “None” and “All” represent two extreme cases, “None” indicated that there was no omental metastasis in all patients, and the clinical net benefit of omental resection was zero; “All” indicate that all patients have omental metastasis, and the clinical net benefit gradually decreases with the increase in the threshold probability. The findings revealed that all three prediction models had clinical decision-making implications. When the threshold probability was between 0 and 0.6, the CP model had better net clinical benefits than the CFP and RSP models. The same finding holds for the test and validation cohorts (Fig. 6D,F) and the CP model still had better net clinical benefits.

## Discussion

It is very difficult to accurately assess the omental metastasis of GC before surgery. In this study, we applied CT radiomics for the first time to the prediction of omental metastasis in GC. Through multivariate logistic regression, we found that CA125, clinical N stage, and radiomics score were independent risk factors for omental metastasis of GC. Therefore, we developed and validated a novel radiomics nomogram for the preoperative prediction of omental metastases status in patients with LAGC.

The prediction model we established has high accuracy in both training and test cohorts, and it was also externally validated. The CP model outperformed the CFP model and the RSP model in all three cohorts. These showed that combining radiomics and clinical features could increase the diagnostic efficacy of the predictive model. In the test cohort, the prediction model had an AUC of 0.836, a sensitivity of 0.917, and a specificity of 0.864. The high sensitivity helps to screen out patients with omental metastasis as much as possible. In addition, we also drew a decision curve for the prediction model, and the results of the decision curve showed that the outcome showed by the prediction model had a greater clinical benefit than the outcome of total omentectomy or omentum preservation. Our prediction model, which combined radiomics features with independent clinical risk features (CA125, clinical N stage), may contribute to the noninvasive and individualized preoperative identification of higher-risk patients with omental metastasis and has crucial clinical significance for the selection of surgical methods. In addition, we also drew a dynamic nomogram and did not need to measure anything manually, making applying the prediction model more convenient.

Radiomics has been widely applied in the research of solid tumors, such as lung cancer, breast cancer, GC, and colorectal cancer^24–27^. In addition, at the molecular level, radiomics was also used to study immune cell infiltration and to evaluate immunotherapy sensitivity^28^. In GC, radiomics was previously used to predict lymph node metastasis, N stage, the efficacy of neoadjuvant chemotherapy, postoperative local recurrence, long-term survival, and so on^29–32^. Dong used CT radiomics to predict the number of lymph node metastases in advanced GC, and the radiomics prediction model showed good accuracy with a c-index of 0.821^30^. Similarly, Wang used CT radiomics to predict whether GC had lymph node metastasis, and showed very good accuracy in the training and test cohorts, with AUC values of 0.886 and 0.881, respectively^31^. CT radiomics still plays an important role in predicting the local recurrence of GC after radical resection, and the AUC value of the radiomics prediction model reached 0.89^32^. The above studies demonstrated the important potential value of radiomics in building predictive models. In our study, The CP model for the omental metastases status of GC likewise exhibited great accuracy, with an AUC value of 0.871. In the test and validation cohorts, the combined prediction model had AUC values of 0.836 and 0.779, respectively.

At present, there are no studies on the prediction of LAGC omental metastasis, and there are only a few related studies on peritoneal metastasis of GC. Liu used CT radiomics to predict peritoneal metastases in LAGC, and the results showed that the AUC values of the predictive models in the training and test cohorts were 0.741 and 0.724, respectively^19^. The AUC values of our omental metastasis prediction model in the training and test cohorts were 0.871 and 0.836, respectively. Compared with Liu's study, we have the advantage of including a larger number of patients, a larger AUC value, and a higher accuracy rate. In addition, we performed external validation, with the AUC value of the combined prediction model in the validation cohort being 0.779. This reflects the fairly good robustness of the prediction model. The reasons for the better performance of our predictive model may be as follows: first, we selected contrast-enhanced CT images in the arterial phase; features extracted from arterial phase CT images seem to perform slightly better than those extracted from the portal phase^33,34^. Wang used features extracted from arterial phase CT images to predict lymph node metastasis in GC with an AUC value of 0.821^12^. Second, Among the radiomic features extracted in this study, 9 features were significantly associated with omental metastases. After multivariate logistic regression analysis, 4 features were selected to calculate the radiomics scores; the purpose of the multivariate logistic regression was to further select features to avoid overfitting the prediction model. Consequently, the prediction model of omental metastasis showed a favorable predictive capability in both cohorts.

Our study included systemic immune-inflammation indices such as NLR and PLR as clinical risk factors for omental metastasis. This was because tumorigenesis and tumor progression was closely related to inflammation, and inflammatory cells promote the proliferation, angiogenesis, and invasion of cancer cells^35^. Neutrophils promote tumor cell proliferation, invasion, and metastasis by changing the tumor microenvironment and secreting inflammatory mediators^36^. Platelet activation was a chemoattractant that induces the metastasis of cancer cells^37^. Lymphocytes were an important component of the cytotoxic immune response, which inhibits the proliferation and invasion of cancer cells through cytokine-mediated cytotoxicity^38^. Therefore, the systemic immune-inflammation index was widely used to predict the survival of malignant tumors^39,40^. Through univariate and multivariate logistic regression, we did not find that NLR and PLR were independent risk factors for omental metastasis. The reason for this differential result may be due to insufficient sample size. As one of the established tumor markers, CA125 was more reliable than the other markers (CT, other serum tumor markers) in the diagnosis of peritoneal metastasis^41^. When the CA125 level was at a cutoff value of 35 U/ml, the sensitivity was 39.4%, the specificity was 95.7%, and the diagnostic accuracy was 90.8%^42^. Similarly, we found that CA125 was an independent risk factor for omental metastasis. CA125 has a weighted score of 0 to 20 in the nomogram of the CP model, a weighted score of 0 to 50 for the clinical N stage, and a weighted score of 0 to 100 for the radiomic score. This indicated that the radiomic score we created played a crucial role in predicting omental metastases.

In this study, the patients from the First Affiliated Hospital of Nanchang University were randomly divided into a training cohort and a test cohort to ensure the consistency of baseline data in both cohorts and to promote the reliability of the conclusions. Furthermore, we used an independent validation cohort for external validation, and the prediction model still performed well. However, our study still has several limitations. First, this study was a multicenter retrospective study and further prospective studies are needed to validate it. Second, although our models were internally and externally validated, our data were all from domestic sources. If available, foreign data can be used for further validation. Finally, arterial phase CT images were selected to segment the ROI, and the prediction capability of the features that were extracted from the portal phase and delayed phase CT images remains to be further verified.

This study proved that the CP model demonstrated a better capability to predict omental metastasis than the CFP and RSP models. The prediction model based on CT radiomics features and clinical features has a satisfactory predictive capability for the omental metastasis of LAGC. It has important practical prospects in clinical decision-making.

## Supplementary Information

Supplementary Information 1.

Supplementary Information 2.

Supplementary Information 3.

Supplementary Information 4.

## Data Availability

The other original contributions presented in the study were included in the article/Supplementary Material. For more inquiries can contact the corresponding authors.
