# Supplementary figures and images for: Development and validation of a CT radiomics and clinical feature model to predict omental metastases for locally advanced gastric cancer
Source: Sci Rep. 2023 May 25;13:8442. doi: 10.1038/s41598-023-35155-y (PMC10213037; doi:10.1038/s41598-023-35155-y)

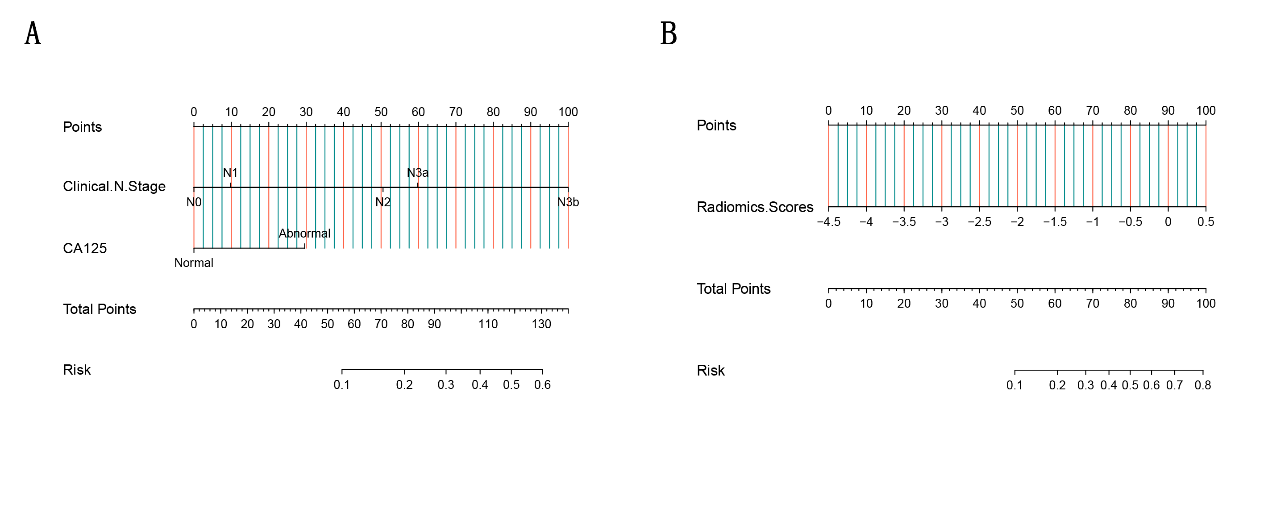


**A**: The predictive nomogram of clinical features; **B**: The predictive nomogram of radiomics scores.

Supplement: Supplementary file 2 — Supplementary Information 2. [file 41598_2023_35155_MOESM2_ESM.docx]
