# Supplementary material for: Development and validation of a CT radiomics and clinical feature model to predict omental metastases for locally advanced gastric cancer
Source: Sci Rep. 2023 May 25;13:8442. doi: 10.1038/s41598-023-35155-y (PMC10213037; doi:10.1038/s41598-023-35155-y)
Supplement: Supplementary file 3 — Supplementary Information 3. [file 41598_2023_35155_MOESM3_ESM.docx]

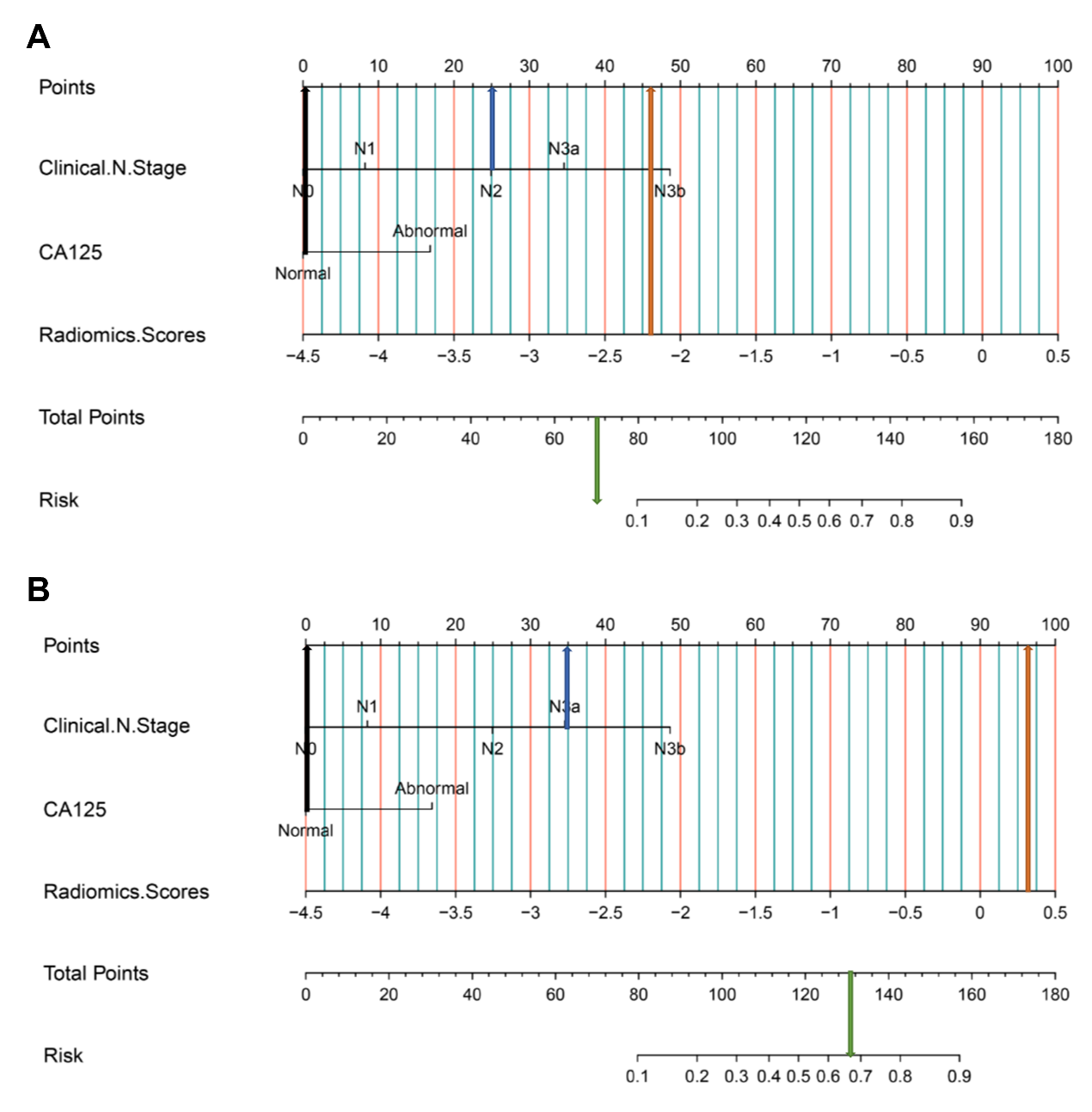


**A**: Case 1, The patient actually had no omental metastasis; male, CA125: normal, clinical N stage: N2, radiomics score:-2.14; The corresponding score of each feature was shown in the figure; Total points= 0 + 25 + 46 = 71, the predictive probability of omental metastasis is less than 0.1. **B**: Case 2, The patient actually had omental metastasis; female, CA125: normal, clinical N stage: N3a, radiomics score:0.3; The corresponding score of each feature were shown in the figure; Total points= 0 + 35 + 96 = 131, the predictive probability of omental metastasis approximately equal to 0.66.
