# Supplementary material for: Development and validation of a CT radiomics and clinical feature model to predict omental metastases for locally advanced gastric cancer
Source: Sci Rep. 2023 May 25;13:8442. doi: 10.1038/s41598-023-35155-y (PMC10213037; doi:10.1038/s41598-023-35155-y)
Supplement: Supplementary file 4 — Supplementary Information 4. [file 41598_2023_35155_MOESM4_ESM.docx]

**Table 1 Characteristics of LAGC patients in the validation cohort**

| **Characteristics** | | **validation cohort (n=104)** |
| --- | --- | --- |
| **Age(years)** | 65.25±11.37 |  |
| **BMI(Kg/m^2^)** | 21.89±2.99 |  |
| **NLR** | 3.53±3.42 |  |
| **PLR** | 210±133.05 |  |
| **Albumin(g/L)** | 41.58±4.41 |  |
| **Tumor size(cm^2^)** | 36.32±38.87 |  |
| **Omental metastases** |  |  |
| No | 91 |  |
| Yes | 13 |  |
| **Gender** |  |  |
| Male | 72 |  |
| Female | 32 |  |
| **CT-reported LN status** |  |  |
| LN (−) | 54 |  |
| LN (+) | 50 |  |
| **CEA** |  |  |
| Normal | 73 |  |
| Abnormal | 31 |  |
| **CA125** |  |  |
| Normal | 75 |  |
| Abnormal | 29 |  |
| **CA19-9** |  |  |
| Normal | 98 |  |
| Abnormal | 6 |  |
| **Borrmann classification** |  |  |
| I | 4 |  |
| II | 35 |  |
| III | 61 |  |
| IV | 4 |  |
| **Tumor location** |  |  |
| Proximal third | 28 |  |
| Middle third | 28 |  |
| Distal third | 47 |  |
| Complete stomach | 1 |  |
| **Clinical T stage** |  |  |
| cT3 | 69 |  |
| cT4a | 15 |  |
| cT4b | 20 |  |
| **Clinical N stage** |  |  |
| N0 | 25 |  |
| N1 | 25 |  |
| N2 | 19 |  |
| N3a | 27 |  |
| N3b | 8 |  |
| **Radiomics scores** | -2.17±0.72 |  |
